# Associations of sedentary time and self-reported television time during pregnancy with incident gestational diabetes and plasma glucose levels in women at risk of gestational diabetes in the UK

**DOI:** 10.1186/s12889-019-6928-5

**Published:** 2019-05-15

**Authors:** Janelle M. Wagnild, Kim Hinshaw, Tessa M. Pollard

**Affiliations:** 1 Department of Anthropology, Durham University, Dawson Building, South Road, Durham, DH1 3LE UK; 2 Department of Obstetrics, Sunderland Royal Hospital, Kayll Road, Sunderland, SR4 7TP UK

**Keywords:** activPAL, Sedentary time, Television time, Gestational diabetes, Pregnancy, Glucose, Prospective study

## Abstract

**Background:**

Sedentary time is associated with increased risk of type 2 diabetes, but the association between objectively measured sedentary time and incident gestational diabetes mellitus (GDM) has not been tested. The purpose of this paper is to test associations between objectively measured sedentary time and self-reported television time during pregnancy with incident GDM and plasma glucose levels among women at high risk for GDM.

**Methods:**

At 20 weeks’ gestation, pregnant women (*n* = 188) in the North East of England with a risk factor for GDM wore an activPAL accelerometer and reported their usual television time. Participants underwent a standard oral glucose tolerance test at 24–28 weeks’ gestation. Regression analyses were used to test for associations of total and prolonged sedentary time, breaks in sedentary time, and television time with GDM and fasting and 2-h glucose levels. Interaction terms were applied to examine whether the association between each indicator of sedentary time and glucose levels differed by GDM status.

**Results:**

Total sedentary time (hours/day) was not associated with incident GDM (OR 1.00 (95%CI 1.00, 1.01)). The association between total sedentary time and glucose levels depended on GDM status: sedentary time was associated with fasting (β = 0.16 (95%CI 0.01, 0.31)) and 2-h (β = 0.15 (95%CI 0.01, 0.30)) glucose levels for those without GDM, while breaks in sedentary time were associated with lower fasting (β *= −* 0.55 (95%CI – 0.92, − 0.17)) and 2-h (β = − 0.40 (95%CI - 0.77, − 0.03)) glucose levels for those with GDM. Prolonged sedentary time was associated with higher fasting glucose levels regardless of GDM status (β 0.15 (0.01, 0.30)). Television time was associated with development of GDM (OR 3.03 (95%CI 1.21, 7.96)) but not with plasma glucose levels.

**Conclusions:**

This is the first study to test associations between posture-based measures of sedentary time during pregnancy and GDM and glucose levels. The findings presented here suggest the possible importance of minimizing or breaking up sedentary time for the management of glucose levels during pregnancy, at least among women at high risk of GDM. Further research is needed to understand the different roles of total sedentary time and television time in the development of GDM.

## Background

A recent meta-analysis found a weak but significant association between total sedentary time, defined as time spent sitting or reclining with low energy expenditure (≤1.5 metabolic equivalents) during waking hours [1], and incidence of type 2 diabetes [2]. Total sedentary time has also repeatedly been found to be associated with indicators of poor glucose metabolism, including increased fasting glucose [3] and raised 2-h plasma glucose levels [4–6] as well as increased insulin levels [3] and indicators of insulin resistance [7] in cross-sectional studies of adult populations. Where studies have controlled for moderate-to-vigorous physical activity (MVPA) and BMI or waist circumference, these associations have persisted [2, 4, 5].

The way in which sedentary time is accumulated throughout the day has been suggested to have additional impact. For example, experimental studies have indicated that breaking up periods of prolonged sitting is associated with reduced postprandial glucose levels [8, 9]. However, the effect of prolonged sitting and breaks in sedentary time has been inconsistent in observational studies, with some reporting that breaks in sedentary time improve glucose levels [10, 11] but others reporting no association [5, 7]. Taken together, the evidence suggests that total sedentary time is associated with type 2 diabetes and with poorer glucose regulation among the general adult population, and this link may be modified by the way in which sedentary time is accumulated throughout the day.

The most commonly assessed sedentary behaviour is television time. Patterson et al. [2] reviewed studies of links between television time (after controlling for physical activity) and incidence of type 2 diabetes and found a stronger relationship than for total sedentary time. Biswas et al.’s meta-analysis [12] also reported a significant association between television time and type 2 diabetes incidence after adjusting for physical activity. Thus, the relationship of television time with incidence of diabetes and glucose metabolism is of particular interest.

There is limited evidence on links between total sedentary time or television time and gestational diabetes mellitus (GDM) incidence or glucose metabolism during pregnancy. As GDM is one of the most common pregnancy complications in countries such as the UK and the US, and it is associated with further pregnancy complications including fetal macrosomia and shoulder dystocia [13], it is important to understand whether physical behaviours are associated with GDM risk. No studies to date have tested associations between objectively measured sedentary time and incidence of GDM. Two studies reported no association between self-reported total sitting time [14] or television time [14, 15] and incident GDM, while one study [16] reported that time spent sitting at home (including watching television) was positively associated with incident GDM among 11,450 Chinese women. Objective measures have been used to test associations between sedentary time and glucose metabolism [17–19] and insulin sensitivity [17, 20] during pregnancy. No studies reported significant associations, but it is important to note that the methods used in these studies estimated sedentary time based on non-movement without a measurement of posture, which is a key distinguishing factor in the measurement of sedentary time. The associations between the way in which sedentary time is accumulated (i.e., prolonged sedentary time, breaks in sedentary time) and glucose metabolism during pregnancy have also not been examined.

This paper is the first study to use an accelerometer that can detect posture (the activPAL) to test prospective associations between objectively measured total sedentary time, development of GDM, and glucose levels among pregnant women in the UK who have a risk factor for GDM. We also set out to test associations between self-reported television time during pregnancy and GDM and glucose levels.

## Methods

### Participants

The target sample size was calculated in 2015 using G*Power [21] based on incident gestational diabetes as the key outcome variable. The required sample size at recruitment was 326 with significance set at 0.05, power at 0.80, and the effect size as OR 1.73, derived from a meta-analysis of studies that included a variety of measures sedentary time [22]. The calculated sample size anticipated a drop-out rate of 30%.

Participants were recruited from two antenatal clinics within hospitals in the North East of England when they attended for their 12-week ultrasound scan between February and August 2017. All participants had to have at least one risk factor for GDM to be eligible for the study (BMI ≥30 kg/m^2^, first-degree relative with diabetes, previous GDM, minority ethnic origin with a high prevalence of diabetes (i.e., South Asian, Black Caribbean), or previous macrosomic baby (≥4.5 kg) [23]). Additional inclusion criteria included that participants were at least 18 years old, pregnant with only one baby, fluent in English, and did not have pre-existing diabetes (because, by definition, one who has pre-existing diabetes cannot develop GDM). All participants provided written consent prior to engagement in any research activities. Ethical approval was provided by the NHS (REC reference: 16/SC/0355).

### Measurement of sedentary time

Participants were asked to wear an activPAL3 (PAL Technologies, Glasgow, UK) continuously for seven days during their second trimester. The activPAL has been validated for the measurement of total sitting time [24] and the detection of transitions from sitting to standing (‘breaks’) [25] in free-living contexts. The activPAL was fitted immediately following the participant’s 20-week anomaly ultrasound scan, upon confirmation from the sonographer that no fetal problems were detected. Prior to fitting, the device was covered with a nitrile sleeve and piece of waterproof Tegaderm to allow for a continuous wear protocol. The activPAL was affixed by a trained member of the research team to the anterior midline of the participant’s right thigh using an 8x10cm piece of Tegaderm. Verbal and written instructions for use were given to each participant; participants were instructed to leave the device on for 24-h per day, removing it only if the leg was going to be fully submerged underwater (e.g., swimming and bathing) or if skin irritation occurred. Participants were instructed to reaffix the activPAL immediately following any periods of removal (on the opposite thigh, if necessary, unless skin irritation was too severe) using additional pieces of Tegaderm that were provided. Participants were also given diaries to record any instances of removal, as well as the start and stop times of all night-time sleep. At the end of the wear period, the devices were collected from participants at their homes or workplaces, or postal arrangements were made as needed. Accelerometry data sets were considered valid if participants provided at least four complete (24-h) days of measurement [6]. We did not require that one of the four days be a weekend day, although 97% of participants who provided four days of measurement provided data for at least one weekend day.

After downloading the raw data from the devices into EventsXYZ files, the activPAL data were processed using a validated automated algorithm in STATA [26] using the algorithm’s default criteria for identifying sleep and non-wear. These criteria defined the main bout of sleep as a continuous bout of non-movement lasting ≥5 h, or ≥ 2 h of non-movement that was also the longest per noon-to-noon 24-h period. Bouts on either side of the main sleep bout were also classified as sleep if they met any or all of the following three criteria: non-movement bouts lasting ≥2 h, ≥30 min non-movement with ≤20 steps, or only postural changes without steps. Days were considered invalid if one posture accounted for ≥95% of waking wear, if there were < 500 steps in a day, or if there were < 10 h of waking wear. The day of fitting or removal were also manually classed as invalid even if they contained > 10 h of waking wear, thus only 24-h days of wear were included in analyses. In accordance with best practice for activPAL data processing [27], heatmaps were generated to visually cross-check the algorithm’s identification of sleep start and stop times against participants’ sleep diaries; in cases of large disparity (i.e., at least an hour’s difference between the two), the algorithm’s sleep identification was manually corrected to align with the diary.

Measurements of total sedentary time (hours per day), prolonged sedentary time (uninterrupted sedentary time (hours per day) accumulated in bouts lasting ≥30 min [6]), breaks in sedentary time (number of sit-to-stand transitions), and time spent stepping (hours per day) were based on the activPAL’s default outputs and were calculated as the sum of each variable on valid days, divided by the number of valid days.

At the time of accelerometer fitting (20 weeks’ gestation), participants were also asked to report the amount of time they usually spent watching television per day in the second trimester (none, < 30 min, 30 min to less than 2 h, 2 h to less than 4 h, 4 h to less than 6 h, ≥6 h). Responses were dichotomized as less than or ≥ 2 h per day [15].

### Measurement of glucose levels and confirmation of GDM

Due to an increased risk status for GDM, participants had a standard 2-h 75 g oral glucose tolerance test (OGTT) after an overnight fast at between 24 and 28 weeks’ gestation as part of their routine pathway of care, providing fasting plasma glucose levels, 2-h glucose levels, and confirming cases of GDM. Twelve eligible participants did not have the OGTT due to health reasons that precluded the glucose test; thus for these participants, no fasting or 2-h glucose values are available, but it is known whether they developed GDM during the pregnancy (four of them did) through other forms of monitoring. GDM was diagnosed in accordance with NICE guidelines (fasting glucose ≥5.6 mmol/litre and/or 2-h ≥ 7.8 mmol/litre) [23].

### Covariates

Participants provided basic details about themselves on an enrolment form after giving consent at their 12-week scan, including whether they had been diagnosed with GDM before. Participants’ BMI recorded at their booking appointment was extracted from medical records.

### Statistical analyses

All statistical analyses were conducted using R. Fasting and 2-h glucose values were positively skewed and were thus log-transformed for all analyses which resulted in a normal distribution. Independent t-tests and chi-square analyses were used to compare characteristics of those who did and did not withdraw from the study, as well as to compare those who did and did not provide valid accelerometry data. Logistic regression was used to test associations with gestational diabetes diagnosis, and linear regression was used to test associations with fasting and 2-h glucose. Although the study took place at two study sites, mixed models were not used because two sites is too few to robustly estimate the random effect, thus recruitment site was included as a control variable in all analyses. All models additionally controlled for age, BMI, and time spent stepping as measured by the activPAL; waking wear time was also controlled in accelerometry models. GDM logistic regression models additionally controlled for previous GDM. Because of the possibility that associations between television time and health outcomes may be confounded by socioeconomic status [28], television time models are shown with and without additional adjustment for household income category. For completeness, we also show the total sedentary time models with and without adjustment for income. Because of abnormalities in glucose metabolism in GDM, we expected the association between sedentary time and glucose levels might be different for those without and without GDM. Thus, we constructed additional linear regression models which included interaction terms between GDM status and centred accelerometry variables; if the interaction was significant and improved the fit of the model significantly based on likelihood ratio tests, estimated marginal means of linear trends were calculated (*emmeans* R package). The estimated marginal means of linear trends for each subgroup were interpreted as significant if the confidence intervals did not cross zero. Complete-case analysis was used for each model; as there are different missing variables within each model, the final sample size per model is specified within the table.

## Results

### Participant characteristics

Of those who were approached to take part in the study and were eligible, 326 consented to take part at the 12-week scan (54.9% response rate). No information about those who declined to take part is available. Sixty-six (20.2%) of those who initially consented withdrew from the study prior to wearing the accelerometer, either through choice (*n* = 46) or because their continued participation was considered inappropriate for medical reasons (e.g., due to fetal anomaly, *n* = 20). Those who were withdrawn (all reasons) were significantly younger than those who were retained in the study (28.6 ± 0.7 vs 30.2 ± 0.3 years, *p* < 0.001); there were no significant differences in other characteristics such as BMI or parity (*p* > 0.05). Of the 260 women who were fitted with the accelerometer, 192 provided valid data sets (≥4 days of 24 h). A substantial proportion of insufficient wear was attributed to skin reactions to the activPAL and/or the dressing with which it was attached; 50 participants indicated either directly to the research team or on their wear diaries that they experienced at least some degree of skin irritation underneath the device. We were unable to retrieve BMI data for four participants; as BMI is a covariate in all models, these participants were excluded from all analyses, resulting in a final analytical sample size of 188.

Characteristics of participants in the analytical sample (*n* = 188) are shown in Table 1.

**Table 1:** Description of the study sample (*n* = 188 unless otherwise specified)

Characteristic	Mean (SD) or n (%)
Age (years)	31.0 (5.1)
BMI (kg/m^2^)	34.8 (5.6)
Parity (*n* = 187)	
Nulliparous	73 (39.0%)
Multiparous	114 (61.0%)
Annual household income category (*n* = 181)	
Less than £20,000	60 (33.1%)
Between £20–40,000	68 (37.6%)
Above £40,000	53 (29.3%)
Family history of diabetes (*n* = 187)	
Yes	57 (30.5%)
No	130 (69.5%)
Ethnicity	
White British	180 (95.7%)
Previous GDM (*n* = 186)	9 (4.8%)
Gestational diabetes diagnosis	31 (16.5%)
Fasting glucose (mmol/litre) (*n* = 176)^a^	4.6 (4.3, 4.9)
2-h glucose (mmol/litre) (*n* = 175)^a^	6.0 (5.2, 7.0)

^a^ Median (interquartile range)

Those who provided valid data sets (n = 188) were significantly older (31.0 ± 5.1 vs 28.3 ± 5.2 years, *p* < 0.001), were more likely to be in the highest household income group (29.3% vs 13.0%, *p* < 0.001), and were more likely to be married/cohabiting (86.6% vs 69.4%, *p* < 0.001) and employed (81.3% vs 65.3%, *p* < 0.01) than those who did not. There was no difference in GDM prevalence between those who did and did not provide valid data sets (*p* = 0.17). All but seven participants who provided valid accelerometry data provided self-reported television time (*n* = 181).

### Descriptive statistics of objectively measured sedentary time and television time

Mean accelerometry variables are shown in Table 2. On average, participants spent 65.1% of waking hours in sedentary time. Sixty-eight (37.6%) of those who provided data on television time reported watching ≥2 h of television per day in the second trimester.

**Table 2:** Accelerometry descriptive statistics (*n* = 188)

Variable	Mean (SD)
Sedentary time (hours/day)	9.56 (1.64)
Prolonged sedentary time (hours/day)	2.38 (0.83)
Breaks in sedentary time (n/day)	52.6 (13.7)
Stepping time^a^ (hours/day)	0.75 (0.64, 1.31)
Waking wear time (hours/day)	14.69 (1.04)

^a^ Median (interquartile range)

### Total sedentary time

Total sedentary time was not associated with risk of developing GDM (Table 3). Total sedentary time had positive but non-significant associations with fasting and 2-h glucose levels (Table 3). Additional adjustment for income did not substantially impact the associations between sedentary time and GDM (OR 1.00 (95%CI 1.00, 1.01)), fasting glucose (β = 0.14 (95%CI -0.02, 0.30)), or 2-h glucose (β = 0.11 (95%CI -0.06, 0.27)).

**Table 3:** Associations of sedentary time with incident GDM and fasting and 2-h glucose levels for the whole sample

	Total sedentary time		Prolonged sedentary time		Breaks in sedentary time^a^			Television time	
	OR (95%CI)	*p*-value	OR (95%CI)	*p*-value	OR (95%CI)	*p*-value		OR (95%CI)	*p*-value
GDM incidence^b^ (*n* = 186)	1.00 (1.00. 1.01)	0.24	1.23 (0.74, 2.04)	0.43	1.00 (0.97, 1.03)	0.98	(*n* = 177)	3.03 (1.21, 7.96)	0.02
	β (95%CI)		β (95%CI)		β (95%CI)			β (95%CI)	
Fasting glucose^c^ (mmol/L) (*n* = 175)	0.12 (−0.03, 0.28)	0.13	0.15 (0.01, 0.30)	0.04	−0.12 (− 0.27, 0.04)	0.13	(*n* = 166)	0.12 (− 0.04, 0.27)	0.13
2-h glucose^c^ (mmol/L) (*n* = 174)	0.11 (− 0.05, 0.27)	0.17	0.07 (− 0.08, 0.22)	0.39	0.06 (− 0.10, 0.22)	0.47	(*n* = 165)	0.05 (− 0.11, 0.20)	0.57

All models adjusted for age, BMI, stepping time, and recruitment site; accelerometry models additionally adjusted for waking wear time

^a^Additionally adjusted for total sedentary time

^b^Additionally adjusted for previous GDM

^c^Log-transformed

The interaction terms between sedentary time and GDM status in relation to fasting and 2-h glucose were significant (*p* < 0.05) and near-significant (*p* = 0.06), respectively. Estimated marginal means of linear trends were applied to the linear regression model which indicated that, for those who did not have GDM, sedentary time was significantly associated with fasting (β = 0.16 (95%CI 0.01, 0.31), SE = 0.08) and 2-h glucose (β = 0.15 (95%CI 0.01, 0.30), SE = 0.07). Sedentary time was not significantly associated with fasting (β = − 0.21 (95%CI-0.50, 0.09), SE = 0.15) or 2-h glucose (β = − 0.15 (95%CI -0.43, 0.14), SE = 0.14) among those with GDM.

### Prolonged sedentary time

Prolonged sedentary time was not significantly associated with risk of developing GDM (Table 3). Prolonged sedentary time had a significant, positive association with fasting glucose but not 2-h glucose (Table 3). The interaction terms between GDM status and prolonged sedentary time in relation to fasting and 2-h glucose levels were not significant (both *p* > 0.05).

### Breaks in sedentary time

Breaks in sedentary time were not significantly associated with risk of developing GDM (Table 3). Breaks in sedentary time were not significantly associated with fasting or 2-h glucose levels (Table 3). The interaction terms between GDM status and breaks in relation to fasting and 2-h glucose were significant (both *p* < 0.05). Estimated marginal means of linear trends indicated that breaks were associated with significantly lower fasting glucose (β *= −* 0.55 (95%CI –0.92, − 0.17), SE = 0.19) and lower 2-h glucose (β = − 0.40 (95%CI -0.77, − 0.03), SE = 0.19) among those with GDM. Breaks had no significant effect on fasting (β = − 0.05 (95%CI -0.20, 0.09), SE = 0.07) or 2-h glucose (β = 0.13 (95%CI -0.01, 0.26), SE = 0.07) among those without GDM.

### Television time

Television time (less than or ≥ 2 h per day) was significantly associated with incident GDM (Table 3) The association between television time and GDM remained significant after additional adjustment for household income category (OR 2.93 (95%CI 1.15, 7.89), *p* = 0.03).

Television time was not significantly associated with fasting glucose levels (Table 3); additional adjustment for household income had a negligible effect (β = 0.11 (95%CI -0.05, 0.26), *p* = 0.18). Television time was also not associated with 2-h glucose levels (Table 3); additional adjustment for household income did not substantially affect the association (β = 0.05 (95%CI -0.11, 0.21), *p* = 0.51). Interaction terms between GDM status and television time in relation to fasting and 2-h glucose levels were not significant (both *p* > 0.05).

## Discussion

This study is the first to test an association between objectively measured sedentary time and incident gestational diabetes and is the first to use a posture-based measure of sedentary time with a prospective study design. In this sample, objectively measured sedentary time in the second trimester of pregnancy was not associated with the development of GDM. The effect of objectively measured sedentary time on glucose levels depended on GDM status. Total sedentary time was associated with increased fasting and 2-h glucose levels among those *without* GDM, while breaks in sedentary time were associated with lower fasting and 2-h glucose levels for those *with* GDM. Prolonged sedentary time was associated with higher fasting glucose levels regardless of GDM status. Television time was significantly associated with incidence of GDM but was not associated with glucose levels.

In this sample, there was no association between total sedentary time and incident GDM. While the sample size in the final GDM model was smaller than the power calculation had indicated was necessary, the effect size in this sample was effectively zero (OR 1.00 (95%CI 1.00, 1.01)), suggesting that sample size did not affect our ability to detect a significant effect. Two recent studies have reported a similar non-significant effect size: the only study to our knowledge that has tested the association between objectively (Actigraph) measured sedentary time and incident type 2 diabetes using a prospective study design (OR 0.95 (95%CI 0.79, 1.15)) [29], and a meta-analysis [2] summarizing evidence of prospective links between total sedentary time (mostly self-reported) and incident type 2 diabetes (RR 1.01 (95%CI 1.00, 1.01)). Larger effect sizes have been reported for the association between objectively measured sedentary time and type 2 diabetes, ranging from OR 1.22 (95%CI 1.13, 1.32) [30] to OR 2.19 (95%CI 1.77, 2.70) [31]; however, these estimates are derived from cross-sectional associations which cannot rule out the possibility of reverse causality (i.e., those with diabetes may sit more because of their diabetes). Further research using prospective study designs to examine the effects of objectively measured sedentary time (particularly accounting for posture) in the development of diabetes is necessary.

This study is the first to find an association between objectively measured sedentary time and glucose levels during pregnancy, although this association was only seen among those who did not have GDM. Three other studies that tested an association between objectively measured total sedentary time and glucose levels during pregnancy [17–19] reported no associations. However, these studies used waist-worn accelerometers with a waking wear protocol; such devices are limited in their ability to differentiate sitting from standing [32, 33], and waking wear protocols are likely to miss the end of the waking day due to non-wear [34], which is often the period of the day with the highest sedentary time [35, 36]. These studies also pooled those with and without GDM in their analyses. In the present study, only the effect of prolonged sedentary time on fasting glucose was seen in pooled analyses, suggesting that associations between total sedentary time and glucose levels may only exist among those without GDM. While an understanding of the biological mechanisms that may drive the association between sedentary time and glucose regulation (in pregnancy and in general) is not well developed, evidence from animal models suggests the lack of skeletal muscle contraction during sedentary time could result in reduced expression of GLUT-4, a transport protein involved in the uptake of glucose from the blood [37].

Breaking up sedentary time was associated with improved glucose levels for those with GDM. This is consistent with evidence suggesting that breaks in sedentary time have been associated with improved glucose regulation among those with type 2 diabetes in free-living contexts [38, 39]. The finding that breaks in sedentary time were beneficial only among those with GDM aligns with experimental evidence suggesting that breaking up sedentary time may particularly improve postprandial glucose levels among those with higher insulin resistance [40] or lower cardiorespiratory fitness [41].

Higher television time in the second trimester was associated with a higher likelihood of developing GDM. Two other studies have tested the association between television time and GDM and reported no association [14, 15]. It is possible that this discrepancy reflects the fact that our sample included only women considered to be at high risk of gestational diabetes, although the incidence of GDM (16.1%) in our sample was similar to that in Padmapriya et al.’s sample of women in Singapore (18.6%) [14].

The effect size for the association between television time and GDM was much larger (OR 3.03) than the effect size for the association between total sedentary time and GDM (OR 1.00) in this sample. This is consistent with Patterson et al.’s [2] meta-analysis results which indicated that the effect size of television time in relation to type 2 diabetes incidence was larger than the effect size of total sedentary time. It has been suggested that television time might be a specific sedentary behaviour whose effect is particularly detrimental [42], perhaps due to an association with snacking behaviours [43] or its potentially prolonged nature, which may have pronounced effects on glucose metabolism [9]. However, our findings do not lend support to the latter suggestion as prolonged sedentary time (measured by the activPAL) was not associated with GDM incidence. The effects of television time might also be confounded by socioeconomic position [28], as television time tends to be higher among those in lower socioeconomic positions compared to those in higher socioeconomic positions [44, 45]. Although the association between television time and GDM persisted after controlling for household income in this sample, income category is not a comprehensive indicator of socioeconomic circumstances. More longitudinal research is needed to improve our understanding of these associations.

This study has several strengths, including the objective measurement of sedentary time using a device that can distinguish posture (activPAL) and detect breaks in sedentary time using a 24-h wear protocol that captures sedentary time throughout the entire day; a prospective study design; and measurements of total sedentary time and self-reported television time within the same cohort. However, this study is not without limitations. While this study had a prospective design, the span of time between the measurement of sedentary time and GDM diagnosis was short (between 4 and 8 weeks). However, based on evidence to suggest that patterns of sedentary time may change across trimesters of pregnancy [46], it was necessary to measure both sedentary time and glucose levels in the same trimester to minimise variability. Television time was self-reported (as is standard practice), which may lead to measurement error. The findings from this study are based on a sample with a risk factor for GDM, thus the results presented here may not necessarily extend to the general pregnant population.

## Conclusion

The findings of this study suggest that reducing total sedentary time and breaking up sedentary time may be a strategy for managing glucose levels, at least among women at high risk of gestational diabetes during pregnancy. This is an area requiring further research. Higher television time during the second trimester was associated with greater incidence of GDM but was not associated with glucose levels. Greater understanding of the mechanisms to explain the association of television time with gestational diabetes is needed. Additional studies that use prospective designs and thigh-worn accelerometers are needed to further our understanding of the role of sedentary time in the development of diabetes and other poor cardiometabolic health outcomes.

## Abbreviations

GDM: Gestational diabetes mellitus
MVPA: Moderate to vigorous physical activity
